# Personalia

**Published:** 1914-01-10

**Authors:** 


### PERSONALIA.

In recognition of their services as Presidents of Sections of the Congress of the British Royal Institute of Public Health in Paris in May last the French Government have conferred the honour of the diploma of "Officier de l'Instruction Publique" upon Sir Thomas Oliver, M.D., Sir Ronald Ross, M.D., Professor E. W. Hope, M.D., and Professor Georges Dreyer, M.D.

Mrs. Snelling, of Southfleet, has been elected chairman of the Ladies' Association of the Gravesend Hospital for the present year, in succession to Mrs. Peyton, who resigned owing to leaving the district. The Association's last and perhaps most successful function for raising funds for the hospital took the form of an "Olde Village Fayre," which was opened by Princess Louise, Duchess of Argyll, in April last, and realised nearly £1,200.

The Committee of Livingstone College announce that Dr. Charles F. Harford, the Principal, is resigning his post next summer. As one who was largely instrumental in founding the College, and as its first Principal, he will be much missed, though he will still remain a member of the Committee. No decision has yet been made as to his successor, but the Committee feel, on personal and other grounds, that the moment is a critical one for the College.

A memorial to the late Mr. John Roderick is to be erected at the General Hospital, Birmingham, of which he was a member of the board for ten years, and to which he left over £100,000 at his death. A large medallion on a marble slab, by Mr. Albert Toft, suitably inscribed, is to be fixed at the entrance to the south-west block, and Mr. Edward Evershed and Mr. Alfred Pointon, Mr. Roderick's executors, have been elected honorary life governors in appreciation of their services.

At Ancoats Hospital, Manchester, recently, an interesting gathering of old and new trustees of the hospital took place. Three gentlemen only of the trustees appointed in 1875 survive, viz., Mr. W. Armitage (chairman of the board of management), the Rev. Canon Nunn, and Mr. C. H. Bazley. These gentlemen having met, and Mr. Bazley resigning his trusteeship, the following gentlemen were appointed trustees to bring the number to the maximum required by the trust-deed :—Mr. N. Armitage, Mr. H. W. Arnold, Mr. P. A. Birley, Mr. Guy Carver, Mr. Oswald Cawley, Mr. Erskine E. Crossley, Mr. W. C. Dunkerley, Mr. A. E. Gaddum, Mr. W. Heap Holland, Mr. John Hall, Mr. J. Howard Helm, Mr. W. Morton Johnson, Mr. Alan J. Maclure, Mr. W. Murray Marsden, Mr. Will Melland, Mr. J. R. Oliver, Mr. J. M. Oliver, and Mr. Sydney O'Hanlon.

